# Long-term effects of intracranial islet grafting on cognitive functioning in a rat metabolic model of sporadic Alzheimer's disease-like dementia

**DOI:** 10.1371/journal.pone.0227879

**Published:** 2020-01-13

**Authors:** Konstantin Bloch, Shay Henry Hornfeld, Shira Dar, Alexey Vanichkin, Irit Gil-Ad, Pnina Vardi, Abraham Weizman

**Affiliations:** 1 Laboratory of Diabetes and Obesity Research, Felsenstein Medical Research Center, Sackler Faculty of Medicine, Tel Aviv University, Petah Tikva, Israel; 2 Laboratory of Biological Psychiatry, Felsenstein Medical Research Center, Sackler Faculty of Medicine, Tel Aviv University, Petah Tikva, Israel; 3 Laboratory of Transplantation, Felsenstein Medical Research Center, Sackler Faculty of Medicine, Tel Aviv University, Petah Tikva, Israel; 4 Research Unit, Geha Mental Health Center, Petah Tikva, Israel; Hungarian Academy of Sciences, HUNGARY

## Abstract

Accumulating evidence suggests that Alzheimer’s disease is associated with brain insulin resistance, as are some other types of dementia. Intranasal insulin administration has been suggested as a potential approach to overcoming brain insulin resistance and improving cognitive functions. Islet transplantation into the cranial subarachnoid cavity was used as an alternative route for insulin delivery into the brain. Recently, the authors showed the short-term beneficial cognitive effect of a small number of intracranially grafted islets in rats with cognitive dysfunction induced by intracerebroventricular administration of streptozotocin (icv-STZ). This was associated with continuous and safe insulin delivery to the rat brain. The current study investigated the long-term effect of intracranial grafting of islets on cognitive functioning in icv-STZ rats. Severe dementia, associated with obesity and cerebral amyloid-β angiopathy, was induced in Lewis inbred rats by icv-STZ. Two months after icv-STZ, one hundred syngeneic islets were transplanted into the cranial subarachnoid space. Two and six months later, cognitive alterations were assessed by Morris water-maze tests. Islet graft survival was evaluated by immunohistochemical and biochemical assays. Improvement was found in spatial learning and memory of grafted rats as opposed to the sham-operated icv-STZ rats. The grafted islets showed intact morphology, intensive expression of insulin, glucagon and glucose transporter 2. Normoglycemic obesity and cerebral amyloid-β angiopathy were found in both grafted and sham-operated icv-STZ rats. In conclusion, islet grafting into cranial subarachnoid space provides an efficient and safe approach for insulin delivery to the brain, leading to a long-term attenuation of icv-STZ-induced cognitive dysfunction.

## Introduction

Accumulating evidence suggests that Alzheimer’s disease (AD)-related dementia may be associated with brain insulin resistance and deficiency [1,2]. Such type of AD-like dementia may be induced in rats by intracerebroventricular administration of a low dose of streptozotocin (icv-STZ) [3–5].

In previous studies, the authors showed that Lewis inbred rats exposed to icv-STZ developed progressive cognitive decline associated with deposition of amyloid-β in blood vessels of the brain and development of microgliosis, astrogliosis and spongiosis in the fimbria-fornix area of the hippocampus. In addition, cognitive dysfunctions in these rats were associated with obesity-related peripheral metabolic abnormalities [6,7]. Insulin can be delivered to the brain using the intravenous, icv, intranasal, ocular or intrathecal routes [8]. Intranasal insulin administration has been suggested as a clinically relevant approach to overcome brain insulin resistance and improve cognitive functions [9,10].

Recently, pancreatic islet grafting into the cranial subarachnoid cavity was used by the authors, as an alternative route for insulin delivery into the brain. In contrast to the intranasal insulin delivery, the grafted islets provide continuous and metabolically regulated insulin delivery to the brain. A small number of islets, grafted into the subarachnoid space of Lewis rats, increased insulin content in the whole brain [11] and in several brain regions (hippocampus and hypothalamus) [12], and attenuated peripheral metabolic abnormalities and cognitive dysfunctions in icv-STZ rats [7]. The current study investigated the long-term (six months) effect of islet transplantation into icv-STZ rats on cognitive functioning and islet graft surviving.

## Materials and methods

### Animals and study design

2.5–3.0 month-old male Lewis (LEW/SsNHsd) inbred rats (Envigo Ltd, Rehovot, Israel) were used in all experiments. The experiments were approved by the Tel Aviv University Animal Care and Use Committee. The rats were maintained on a regular 12-hr dark/light cycle with access to food and water *ad libitum*. Standard chow diet was from Envigo Ltd. The animals were divided into three groups, as follows: 1) intact rats (N = 9); 2) rats exposed to icv-STZ and two months later to subarachnoid grafting of pancreatic islets suspended in the vehicle (STZ-islets rats) (N = 7); and 3) rats exposed to icv administration of streptozotocin (icv-STZ) and two months later to subarachnoid administration of a vehicle (Hank's Balanced Salt Solution, HBSS) only (STZ-sham rats) (N = 4). Two and six months after the islet grafting, cognitive functioning was studied by Morris water maze (MWM) test. At the end of experiments, overdose of carbon dioxide was used for euthanasia. The brains were removed and analyzed by histological and biochemical methods. In all assays, the assessors were blind to the group status (experimental versus control) of the rats.

### Icv administration of STZ

Rats were anesthetized with a mixture of ketamine and xylazine through intraperitoneal administration. Icv injections of STZ dissolved in artificial cerebrospinal fluid (CSF) were carried out as described previously [6]. Briefly, 8 μl STZ (3 mg STZ/kg body weight) were bilaterally injected (4 μl/2 min on each side) in anesthetized rats using a stereotactic apparatus (Stoelting Co., Wood Dale, IL) according to the following coordinates: 0.9 mm posterior to bregma, 1.8 mm lateral to sagittal suture and 3.8 mm ventral.

### Intracranial grafting of pancreatic islets

Donor islets were isolated from the pancreata of LEW/SsNHsd rats by enzymatic digestion, then purified, cultured, quantified in islet equivalent (IEQ) and transplanted as described previously [7]. About 100 IEQ suspended in 15 μl of vehicle were inserted into the subarachnoid space of anesthetized rats through a hole of 1mm diameter, made in the parietal bone with coordinates: 2 mm to the right and 2 mm posterior to the bregma. The incision was closed with surgical suture and disinfected with povidone iodine.

### Morris water maze (MWM) test

Two months and six months after islet grafting, spatial learning and memory were tested as previously described [7]. Briefly, a circular platform was placed 1 cm under the water surface of a water maze circular pool (diameter– 1.8 m; height– 60 cm). Rats performed four trials per day (up to 120 s for each trial), on three consecutive days. The time taken to reach the platform was recorded. If a rat did not find the platform within 120 s, it was manually placed on it for 20 s and the value was considered 120. In addition, motility parameters were analysed. Data were recorded using an automated tracking system (Ethovision 3.1 Noldus Information Technology B.V., Wageningen, The Netherlands).

### Histological analysis of the brain

Paraformaldehyde (4%), perfused via the vascular system, was used for whole fixation of the anesthetized rats. Either two or three rats were perfused in each experimental group. The brains were removed, processed for paraffin sectioning (4μm thick) and stained with hematoxylin-eosin (H&E). Immunohistochemical analyses of the grafted islets were assayed in sagittal sections of the brain, which were incubated overnight at 4°C with either polyclonal guinea pig anti-insulin antibodies diluted 1:200 (Cell Marque, Rocklin, USA), or monoclonal mouse anti-glucagon antibody diluted 1:2000 (Sigma-Aldrich, St Louis, MO, USA), or polyclonal rabbit anti-glucose transporter 2 (Glut2) antibodies diluted 1:150 (Novus Biologicals, Littleton, CO, USA) or monoclonal mouse proinsulin antibodies at the concentration of 10 μg/ml (R&D Systems, Minneapolis, MN, USA). After washing, the sections were incubated for 1 h at room temperature with secondary antibodies diluted 1:400 (Cy^tm3^- conjugated affinity pure rabbit or anti-guinea pig Ig and Cy^tm2^-anti-mouse Ig). All secondary antibodies were purchased from Jackson Immuno Research Laboratories Inc., Baltimore, PA, USA. Nuclei were counterstained with DAPI diluted 1:200. Cerebral amyloid-β depositions were detected immunohistohemically. Briefly, tissue pre-treatment to unmask immunoreactive sites was conducted using incubation with 98% formic acid for 7 min at room temperature. PeroxFree Block (Cell Marque, Rocklin, CA, USA) was used to block the endogenous peroxidase enzymes. The brain sections were incubated overnight at 4°C, with mouse monoclonal anti-amyloid-β antibodies (1:300) obtained from Novus Biologicals (Littleton, CO, USA). Staining was detected using mouse/rabbit PolyScan HRP/DAB detection system (Cell Marque, Rocklin, CA, USA), according to the manufacturer's protocol. Sagittal brain sections of 5XFAD transgenic mice (males, eight months old) were used as a positive control for amyloid-β deposition in the brain. Immunostaining of all tested antigens was negative when the primary antibodies were replaced with antibody diluent or normal serum.

### Brain insulin content

The right cerebral hemispheres were frozen in liquid nitrogen and stored at −80°C. Cold lysisbuffer (4 ml; 100 mM Tris, pH 7.4, 150 mM NaCl, 1 mM EGTA, 1 mM EDTA, 1% Triton X-100, 0.5% sodium deoxycholate) supplemented with protease inhibitors (P2714, Sigma-Aldrich, St Louis, MO, USA) were added to the whole right brain hemisphere and homogenized on ice for 20 seconds, using an electric motor grinder (Polytron). The homogenates were centrifuged at 12,000g for 20 min at 4°C. The supernatants were removed to measure the protein concentration using a BCA kit (Pierce, Rockford, IL, USA). ELISA kits were used to measure rat insulin (Mercodia AB, Uppsala, Sweden) in the brain extracts according to the manufacturer's protocols.

### Glycemia and insulinemia

Non-fasting blood glucose levels were measured in whole blood samples between 9 a.m. and 10 a.m. with a portable glucometer (Accu-Check; Hoffmann La Roche, Basel, Switzerland). An intraperitoneal glucose tolerance test (IPGTT) was performed in all the groups at the end of the experiment. A glucose solution (1 g glucose/kg body mass) was injected intraperitoneally after 6 h fasting. Blood glucose was measured before glucose solution administration and at 15, 30, 60, and 120 mins after glucose injection. Rat serum insulin was measured using ELISA kit (Mercodia AB, Uppsala, Sweden).

### Statistical analysis

Data were analysed using the Statistical Package for the Social Sciences (SPSS) 17.0 software (SPSS Inc., Chicago, IL). Comparisons between the groups for glucose tolerance were performed using two-way repeated measure analysis of variance (RM-ANOVA) followed by the Bonferroni post hoc test. Alterations in brain insulin level were analysed by one way analysis of variance (ANOVA) followed by Tukey's post hoc test. The results were expressed as Mean ± SE and significance was set at p < 0.05. Cognitive functioning in the MWM experiments (escape latency) were evaluated using a Multivariable Cox regression time-to-event approach in SAS® software, as previously described [7]. The MWM motility parameters were analysed using two way RM-ANOVA followed by the Bonferroni post hoc test.

## Results

### Body weight, insulinemia and glucose tolerance

**Fig 1:**
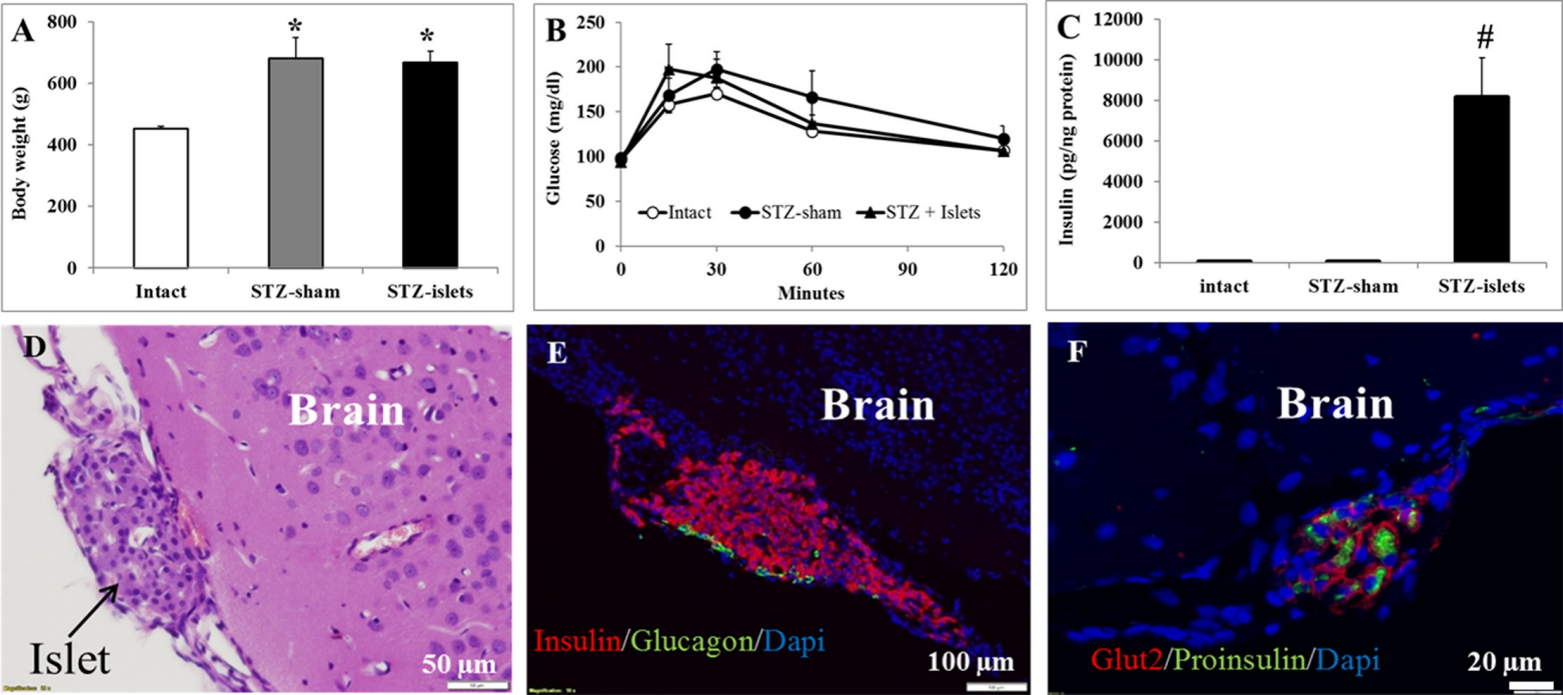
Characteristics of peripheral metabolism and grafted islets in icv-STZ rats. Body weight (A), glucose tolerance (B) and brain insulin content (C). * *p* < 0.0001 versus intact group. ^**#**^ *p*<0.0001 versus intact and STZ-sham groups. Grafted islets located onto the cerebral cortex: H&E staining (D), insulin, glucagon and DAPI staining (E), proinsulin, Glut2 and DAPI staining (F).

One-way ANOVA analysis revealed that at eight months after icv-STZ, the body weights of both non-transplanted (STZ-sham group) and transplanted rats (STZ-islets) were significantly higher than that of intact rats (Fig 1A, S1 Table). At this time point, both STZ-sham and STZ-islets groups expressed hyperinsulinemia (8.95 ± 2.76 ng/ml and 8.72 ± 3.18 ng/ml, respectively) while intact rats showed normal level of serum insulin (0.91 ± 0.31 ng/ml). In spite of the differences in blood level of insulin, all groups showed the physiologically normal range of non-fasting blood glucose levels (90–120 mg/dl) and intact IPGTT (Fig 1B).

### Biochemical and histological analysis of the brain

The insulin levels in the right cerebral hemispheres were measured by ELISA. One-way ANOVA revealed a statistically significant elevation of brain insulin content in STZ-islet rats compared to intact and STZ-sham rats (Fig 1C, S1 Table). Six months after grafting, the islets showed intact morphology (Fig 1D) and intensive staining for insulin and glucagon (Fig 1E) as well as for proinsulin and Glut2 (Fig 1F). Histological analyses did not reveal islet-like structures in the brain of intact and STZ-sham rats.

**Fig 2:**
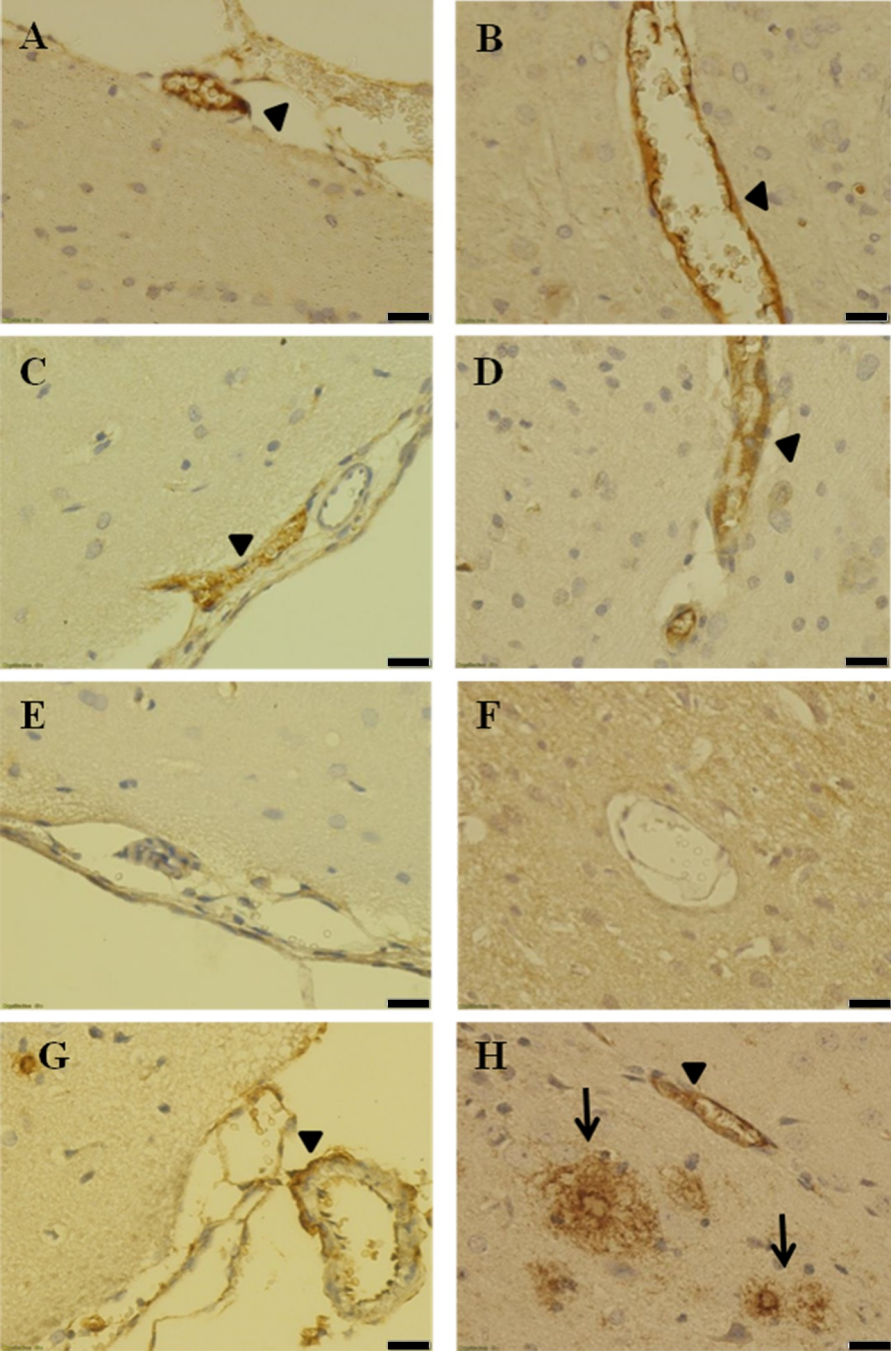
Cerebral amyloid-β angiopathy in icv-STZ rats. STZ-sham (A and B), STZ-islets (C and D), intact (E and F) and 5XFAD mice (G and H). Amyloid-β deposition in intracerebral blood vessels (right panels) and meningeal capillaries (left panels). Head arrows show amyloid-β angiopathy. Arrows show diffuse deposition of amyloid-β in the brain parenchyma of 5xFAD mice. Scale bars = 20 μm.

Amyloid-β depositions were found in the brain of non-transplanted (Fig 2A and 2B) and islet-grafted icv-STZ rats (Fig 2C and 2D), but not in the group of intact rats (Fig 2E and 2F). The amyloid-β was expressed in both intracerebral blood vessels and meningeal capillaries. In contrast, aged 5xFAD transgenic mice, used as a positive control, demonstrated both intensive vascular and diffuse deposition of amyloid-β in the brain parenchyma (Fig 2G and 2H).

### Cognitive functioning

**Fig 3:**
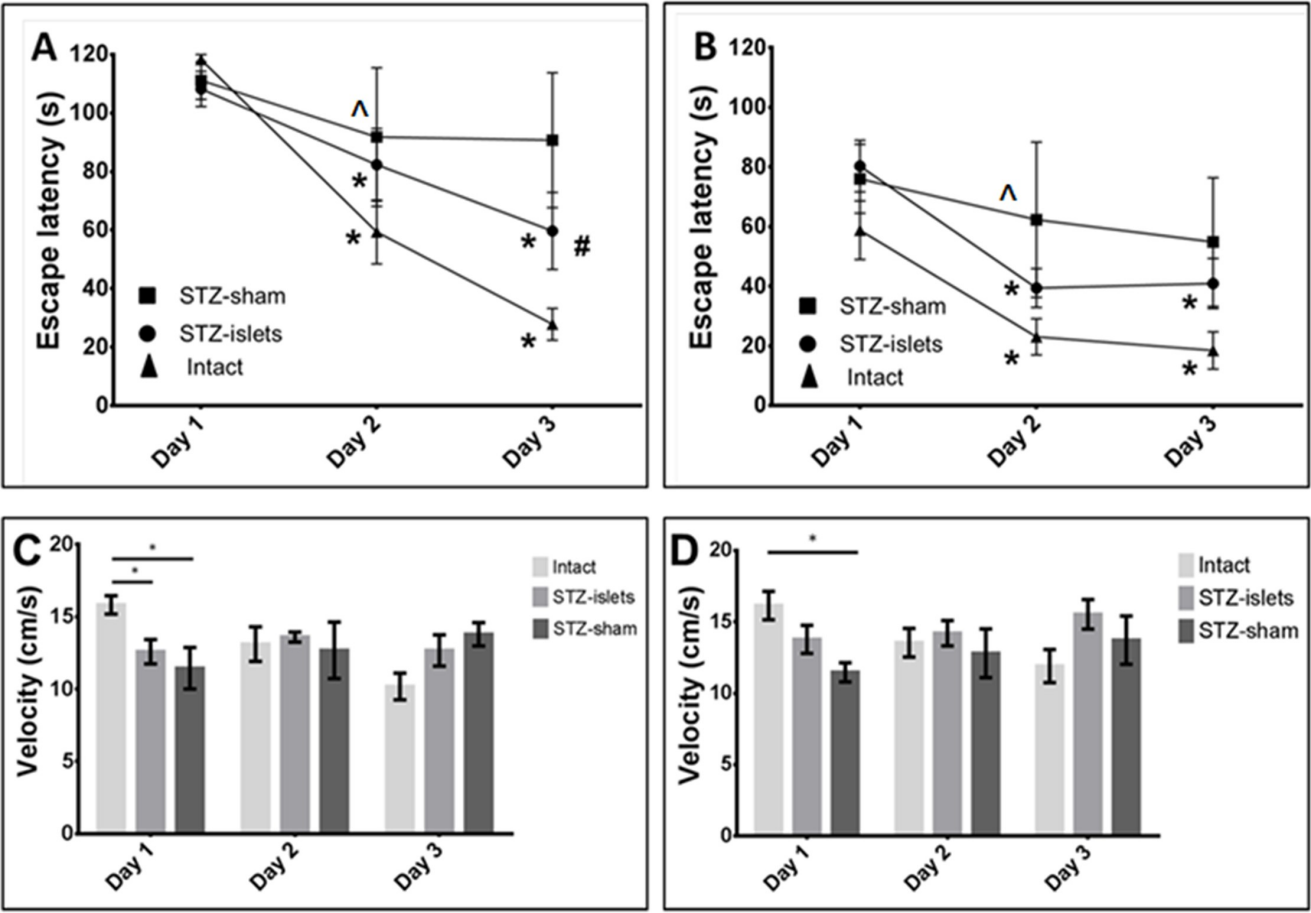
Cognitive functions (MWM test). Escape latency at two months (A) and six months (B) after islet grafting. Velocity at two months (C) and six months (D) after islet grafting.* *p* < 0.0002 compared to day 1. ^**#**^ p < 0.05 compared to STZ-sham rats. **^** p < 0.03 compared to intact rats. Data presented as average ± S E.

Statistical analyses of spatial learning, memory and motility parameters (velocity) in the MWM tests are presented in S2 and S3 Tables and depicted in Fig 3. Two and six months after grafting, STZ-sham rats manifested a profound deficit in spatial learning capacity and memory compared to intact and STZ-islets rats (Fig 3A and 3B). This cognitive impairment in STZ-sham rats was confirmed by statistically non-significant changes in escape latency between first day and consecutive days of the MWM tests. In contrast, STZ-islets and intact rats revealed a better cognitive capacity as reflected by significantly reduced escape latency on day 2 and day 3 compared to day 1 of the MWM tests. Attenuation of cognitive dysfunctions in STZ-islets rats compared to sham-operated animals was also demonstrated by statistically undistinguished differences between escape latency of islet-transplanted and intact rats at day 2 of both MWM tests. Moreover, statistically significant decline in escape latency in STZ-islets rats compared to STZ-sham rats was found on days 3 of the MWM test performed two months after islet grafting.

Analysis of velocity did not reveal statistically significant differences between the STZ-sham and the STZ-islets group in either MWM tests. Reduced velocity of STZ-treated rats compared to intact rats was found only on day 1 of the MWM tests, but did not reach statistically significant differences between the groups at this time point (Fig 3C and 3D).

## Discussion

Progressive cognitive decline is commonly seen in patients with sporadic AD [13]. A transient improvement of cognitive status of patients with AD is very rare [14]. Recently, we demonstrated a progressive cognitive impairment in obese icv-STZ Lewis rats for three months after induction of cognitive decline [6] and showed that the icv-STZ-induced cognitive dysfunction may be ameliorated by intracranial islet grafting [7]. In the current study, we assessed the long-term (six months) therapeutic effect of intracranial grafting of islets on cognitive function in the icv-STZ rats. The exposure of Lewis rats to icv-STZ induced a severe cognitive decline associated with cerebral amyloid-β angiopathy, abdominal obesity and hyperinsulinemia, but did not interfere with the intact peripheral glucose tolerance. The obese rats did not show reduced locomotion (except at first day) in MWM test. Hence, the cognitive decline found in the icv-STZ rats cannot be explained by impaired mobility. The grafted islets improved cognitive functions in icv-STZ rats during six-month post-grafting period, but complete restoration of spatial learning and memory was not achieved.

Another important issue is the long-term survival of the islets grafted into the cranial subarachnoid cavity. Recently, we showed trafficking of viable grafted islets in cerebrospinal fluid (CSF) from the grafted site to remote brain areas such as the olfactory bulb and the lateral ventricles [15]. The main factors perturbing survival of grafted islets are immune rejection and poor supply of oxygen and nutrients. In order to maintain the survival of grafted islets, we used as a grafting site the immunoprivileged subarachnoid cavity filled with CSF, which is a “nourishing liquor” enriched with oxygen, macro- and micronutrients [16]. In addition, continuous flow and CSF-based clearance provide efficient removal of waste metabolites accumulated in the grafted islets. At six-month post-grafting period, grafted islets showed intact morphology, intensive expression of insulin, proinsulin and glucagon, as well as Glut2, which is the main regulator of glucose sensing in islet beta-cells. Another parameter indicating long-term viability of grafted islets is the high concentration of islet-based insulin in the brain of the transplanted rats compared to barely detectable brain insulin in non-transplanted rats. The high insulin content in the brain of STZ-islets group probably depends mainly on insulin content of functioning grafted islets. Taken together, these data indicate that the selected transplantation site enables long-term survival of the grafted islets. It is unclear why the body weight was larger and blood insulin level was higher in icv-STZ treated groups compared to intact rats. Recently, we suggested that the icv-STZ induces hypothalamic abnormalities leading to persistent weight gain and hyperinsulinemia, indicating a metabolic type of dementia [6].

In the present study, we found a moderate accumulation of amyloid-β in the cerebral blood vessels of both non-transplanted and islet-transplanted icv-STZ Lewis rats. Cerebral amyloid-β angiopathy is most commonly detected in Alzheimer's disease. This type of amyloidosis was also found in several animal models of Alzheimer's disease-like dementia [17], including icv-STZ rats [18]. Amelioration of cerebral amyloid-β expression was revealed in mouse models of Alzheimer's disease treated with intranasal insulin [19, 20]. However, a pilot clinical trial showed that the CSF amyloid-β levels did not change significantly in AD patients treated with intranasal insulin compared to placebo [21]. Currently, the role of cerebral amyloid-β in the pathogenesis of this neurodegenerative disorder is still unclear.

There are several limitations in the present study. First, the relatively small number of animals in the various experimental groups. Second, the molecular mechanisms underlying the beneficial effect of intracranial islet transplantation on cognitive functions are still unclear and merit a further investigation.

In conclusion, obese Lewis rats with dementia induced by icv-STZ provide a useful model for investigation of the effectiveness of long-term anti-dementia therapies. In the case of cell therapy approach, cranial subarachnoid cavity appears to be a favorable transplantation site supporting long-term survival of grafted islets and leading to amelioration of cognitive decline in a rat metabolic model of sporadic Alzheimer's disease.

## Supporting information

S1 Table Body weight and brain insulin levels: One-way ANOVA. (DOCX)

S2 Table Multivariable Cox regression analysis for the MWM tests (time to platform). (DOCX)

S3 Table Velocity of the rats in the MWM tests. (DOCX)
