# Supplementary material for: Long-term effects of intracranial islet grafting on cognitive functioning in a rat metabolic model of sporadic Alzheimer's disease-like dementia
Source: PLoS One. 2020 Jan 13;15(1):e0227879. doi: 10.1371/journal.pone.0227879 (PMC6957181; doi:10.1371/journal.pone.0227879)
Supplement: S1 Table — (DOCX) [file pone.0227879.s001.docx]

**S1 Table.** Body weight and brain insulin levels: One-way ANOVA.

| **Test** | **F** | **ANOVA  P Value** | **Turky’s P Value**  Intact vs.  STZ-sham | **Turky’s P Value** Intact vs.  STZ-Islets | **Turky’s P Value**  STZ-Islets vs.  STZ-sham |
| --- | --- | --- | --- | --- | --- |
| Body weight | F_(2,17)_ = 21.558 | P<0.0001 | P<0.0001 | P<0.0001 | N.S. |
| Brain insulin | F_(2,13)_ = 24.459 | P<0.0001 | N.S. | P<0.0001 | P<0.0001 |
|  |  |  |  |  |  |

N.S. - Not significant.
