# Supplementary material for: Long-term effects of intracranial islet grafting on cognitive functioning in a rat metabolic model of sporadic Alzheimer's disease-like dementia
Source: PLoS One. 2020 Jan 13;15(1):e0227879. doi: 10.1371/journal.pone.0227879 (PMC6957181; doi:10.1371/journal.pone.0227879)
Supplement: S2 Table — (DOCX) [file pone.0227879.s002.docx]

**S2 Table.** Multivariable Cox regression analysis for the MWM tests (time to platform).

Two months after islet transplantation:

|  | **Groups** | **Day** | **Point Estimate** | **95% Confidence Limits** | **Z Value** | **P** |
| --- | --- | --- | --- | --- | --- | --- |
|  | Intact vs.  STZ-islets | 2 | 2.126 | 0.593-7.628 | 1.16 | N.S. |
|  | Intact vs.  STZ-sham | 2 | 6.277 | 1.227-32.108 | 2.21 | 0.0274 |
|  | Intact vs.  STZ-islets | 3 | 4.52 | 1.211-17.263 | 2.24 | 0.025 |
|  | Intact vs.  STZ-sham | 3 | 23.002 | 4. 268-123.972 | 3.65 | 0.0003 |
|  | STZ-islets vs.  STZ-sham | 3 | 5.032 | 1.011-25.037 | 1.97 | 0.0484 |
|  | Intact | 1 vs. 2 | 0.015 | 0.000-0.024 | -3.78 | 0.0002 |
|  | Intact | 1 vs. 3 | 0.002 | 0.000-0.024 | -5.13 | <0.0001 |
|  | Intact | 2 vs. 3 | 0.16 | 0.052-0.496 | -3.17 | 0.0015 |
|  | STZ-islets | 1 vs. 2 | 0.187 | 0.044-0.801 | -2.26 | 0.0239 |
|  | STZ-islets | 1 vs. 3 | 0.064 | 0.014-0.295 | -3.53 | 0.0004 |
|  | STZ-sham | 1 vs. 2 | 0.613 | 0.097-3.884 | -0.52 | 0.603 |
|  | STZ-sham | 1 vs. 3 | 0.359 | 0.064-2.004 | -1.17 | 0.243 |

Six months after islet transplantation:

|  | **Groups** | **Day** | **Point Estimate** | **95% Confidence Limits** | **Z Value** | **P** |
| --- | --- | --- | --- | --- | --- | --- |
|  | Intact vs.  STZ-islets | 2 | 6.049 | 0.687-53.262 | 1.62 | N.S. |
|  | Intact vs.  STZ-sham | 2 | 23.078 | 1.411-377.508 | 2.20 | 0.028 |
|  | Intact vs.  STZ-islets | 3 | 14.649 | 1.613-133.018 | 2.38 | 0.017 |
|  | Intact vs.  STZ-sham | 3 | 48.120 | 2.975-778.342 | 2.73 | 0.0064 |
|  | Intact | 1 vs. 2 | 0.050 | 0.014-0.181 | -4.55 | <0.0001 |
|  | Intact | 1 vs. 3 | 0.016 | 0.004-0.067 | -5.74 | <0.0001 |
|  | STZ-islets | 1 vs. 2 | 0.077 | 0.020-0.302 | -3.68 | 0.0002 |
|  | STZ-islets | 1 vs. 3 | 0.061 | 0.016-0.235 | -4.07 | <0.0001 |
|  | STZ-sham | 1 vs. 2 | 0.338 | 0.040-2.844 | -1.00 | N.S. |
|  | STZ-sham | 1 vs. 3 | 0.232 | 0.034-1.572 | -1.50 | N.S. |

N.S. - Not significant.
