# Supplementary material for: Long-term effects of intracranial islet grafting on cognitive functioning in a rat metabolic model of sporadic Alzheimer's disease-like dementia
Source: PLoS One. 2020 Jan 13;15(1):e0227879. doi: 10.1371/journal.pone.0227879 (PMC6957181; doi:10.1371/journal.pone.0227879)
Supplement: S3 Table — (DOCX) [file pone.0227879.s003.docx]

**S3 Table.** Velocity of the rats in the MWM tests.

Two months after islet transplantation:

| **Test** | **F** | **ANOVA  P Value** | **Turky’s P Value**  Intact vs.  STZ-sham | **Turky’s P Value** Intact vs.  STZ-Islets | **Turky’s P Value**  STZ-Islets vs.  STZ-sham |
| --- | --- | --- | --- | --- | --- |
| Day 1 | F_(2,17)_ = 7.075 | P=0.0058 | P=0.0113 | P=0.0257 | N.S. |
| Day 2 | F_(2,17)_ = 0.1258 | P=0.8826 | N.S. | N.S. | N.S. |
| Day 3 | F_(2,17)_ = 3.241 | P=0.0642 | N.S. | N.S. | N.S. |

Two-way RM-ANOVA analysis for evaluation of velocity in the MWM test showed significant interactions (F_(4,34)_ = 5.767; p = 0.0012), therefore we used one-way ANOVA and the Tukey's post-hoc test for each time point. N.S. - Not significant.

Six months after islet transplantation:

| **Test** | **F** | **ANOVA  P Value** | **Turky’s P Value**  Intact vs.  STZ-sham | **Turky’s P Value** Intact vs.  STZ-Islets | **Turky’s P Value**  STZ-Islets vs.  STZ-sham |
| --- | --- | --- | --- | --- | --- |
| Day 1 | F_(2,17)_ = 4.695 | P=0.0238 | P=0.0222 | N.S. | N.S. |
| Day 2 | F_(2,17)_ = 0.3121 | P=0.7360 | N.S. | N.S. | N.S. |
| Day 3 | F_(2,17)_ = 2.503 | P=0.1115 | N.S. | N.S. | N.S. |

Two-way RM-ANOVA analysis for evaluation of velocity in the MWM test showed significant interactions (F_(4,34)_ = 4.922; p = 0.0031), therefore we used one-way ANOVA and the Tukey's post-hoc test for each time point. N.S. - Not significant.
